# The association between pulse wave analysis, carotid-femoral pulse wave velocity and peripheral arterial disease in patients with ischemic heart disease

**DOI:** 10.1186/s12872-021-01859-0

**Published:** 2021-01-13

**Authors:** Nejc Piko, Sebastjan Bevc, Radovan Hojs, Franjo Husam Naji, Robert Ekart

**Affiliations:** 1 Department of Dialysis, Clinic for Internal Medicine, University Medical Centre Maribor, Ljubljanska ulica 5, 2000 Maribor, Slovenia; 2 Department of Nephrology, Clinic for Internal Medicine, University Medical Centre Maribor, Ljubljanska ulica 5, 2000 Maribor, Slovenia; 3 Department of Cardiology, Clinic for Internal Medicine, University Medical Centre Maribor, Ljubljanska ulica 5, 2000 Maribor, Slovenia; 4 Medical Faculty Maribor, University of Maribor, Taborska ulica 8, 2000 Maribor, Slovenia

**Keywords:** Ankle brachial index, Peripheral arterial disease, Arterial stiffness, Atherosclerosis, Myocardial ischemia

## Abstract

**Introduction:**

Functional changes in peripheral arterial disease (PAD) could play a role in higher cardiovascular risk in these patients.

**Methods:**

123 patients who underwent elective coronary angiography were included. Ankle-brachial index (ABI) was measured and arterial stiffness parameters were derived with applanation tonometry.

**Results:**

6 patients (4.9%) had a previously known PAD (Rutherford grade I). Mean ABI was 1.04 ± 0.12, mean subendocardial viability ratio (SEVR) 166.6 ± 32.7% and mean carotid-femoral pulse wave velocity (cfPWV) 10.3 ± 2.4 m/s. Most of the patients (n = 81, 65.9%) had coronary artery disease (CAD). There was no difference in ABI among different degrees of CAD. Patients with zero- and three-vessel CAD had significantly lower values of SEVR, compared to patients with one- and two-vessel CAD (159.5 ± 32.9%/158.1 ± 31.5% vs 181.0 ± 35.2%/166.8 ± 27.8%; *p* = 0.048). No significant difference was observed in cfPWV values. Spearman's correlation test showed an important correlation between ABI and SEVR (r = 0.196; *p* = 0.037) and between ABI and cfPWV (r = − 0.320; *p* ≤ 0.001). Multiple regression analysis confirmed an association between cfPWV and ABI (β = − 0.210; *p* = 0.003), cfPWV and mean arterial pressure (β = 0.064; *p* < 0.001), cfPWV and age (β = 0.113; *p* < 0.001) and between cfPWV and body mass index (BMI (β = − 0.195; *p* = 0.028), but not with arterial hypertension, dyslipidemia, diabetes mellitus or smoking status. SEVR was not statistically significantly associated with ABI using the same multiple regression model.

**Conclusion:**

Reduced ABI was associated with increased cfPWV, but not with advanced CAD or decreased SEVR.

## Introduction

Atherosclerosis is a chronic inflammatory vascular disease, characterized by progressive plaque build-up in the vessel wall, leading to tissue ischemia [1, 2]. It is correlated with different forms of cardiovascular disease (CVD), such as coronary artery disease (CAD), cerebrovascular disease and peripheral arterial disease (PAD) [3]. CVD is one of the leading causes of death in Western countries, contributing to over one-third of deaths in patients aged 35 years or more [4], with men more often affected than women [5].

Traditional risk factors for atherosclerosis and consequently CVD include genetic predisposition, arterial hypertension, dyslipidemia, diabetes mellitus, metabolic syndrome, smoking, a diet rich in fats and psychological stress [6]. Chronic kidney disease (CKD) and albuminuria/proteinuria are independent risk factors for advanced atherosclerosis [7, 8]. Non-traditional risk factors are chronic inflammation, increased levels of lipoprotein(a), hyperhomocysteinemia, and increased vessel wall stiffness [9].

PAD is one of the most prominent forms of CVD and is associated with significant morbidity and mortality [10]. Despite initiatives to improve the recognition and treatment of patients with PAD, the number of people affected and the morbidity continues to rise [11]. By 2010, more than 200 million people worldwide were diagnosed with PAD. Prevalence studies in the United States estimate that 5.9% of Americans over 40 years of age have PAD, with the prevalence even higher in African Americans and Hispanics [12]. In Europe, it is estimated that 5.3% or nearly 40 million people have PAD [13].

PAD can be considered a clinical model of atherosclerosis that combines structural and functional alterations of the vessel wall. A reduced diameter of the arterial lumen is an important structural change and can be assessed by using the ankle-brachial index (ABI) [14]. The ABI is a simple, inexpensive and non-invasive diagnostic tool in establishing the PAD diagnosis in the general population [15]. ABI values of < 0.9 are diagnostic for PAD and can objectively establish the diagnosis of PAD [16]. Reduced ABI has several important prognostic implications given that the presence of symptomatic or asymptomatic PAD is also an indicator of atherosclerosis in other vascular territories, especially coronary arteries [17], increasing the risk of premature mortality due to cardiovascular and cerebrovascular events [18].

Fuctional alterations of the arterial walls are the consequence of changes in the viscoelastic properties of the conduit arteries and can be recognized by using arterial stiffness measurements [14].

Increased arterial stiffness is a hallmark of the aging process and atherosclerosis, including PAD. It is linked to increased velocity of both forward and reflected pulse waves, ultimately causing increased cardiac workload and reduced myocardial perfusion [19]. Arterial stiffness is inherently linked to systolic, diastolic and mean arterial pressures (MAP) [20]. Applanation tonometry is a non-invasive, easily reproducible technique of measuring arterial stiffness. It enables us to perform carotid-femoral pulse wave velocity (cfPWV) and pulse wave analysis (PWA) from which several central hemodynamic parameters can be derived [21].

Patients with PAD experience adverse cardiovascular events more often, which has thus far been mostly attributed to the systemic nature of atherosclerosis and the coexistent presence of atherosclerosis in other vascular territories. Impaired peripheral perfusion can lead to changes in pulse wave reflection and can, therefore, impact myocardial perfusion by itself as well [22]. Due to systemic nature of atherosclerosis, our objective was to determine if there is correlation between peripheral and coronary perfusion, the former determined by ABI and the latter by coronary angiography. Afterwards, our objective was to determine the potential association between ABI, CAD and several arterial stiffness parameters, which are a marker of not only structural, but also functional alterations in vessel walls. Finally, we wanted to assess if the impact of ABI on arterial stiffness is independent of atherosclerosis risk factors, implying an important role of changed pulse wave propagation in understanding the increased cardiovascular risk observed in patients with PAD.

## Methods

### Study population

The study was a cross-section, single-center evaluation of ABI, cfPWV and PWA parameters, conducted at a tertiary referral center (University Medical Centre Maribor, Slovenia). All the data was obtained in a 2 year period, between October 1st, 2017 and October 1st, 2019. 123 non-consecutive Caucasian patients, older than 18 years, who were hospitalized at the Department of Cardiology due to planned, elective coronary angiography (previously positive either cycle ergometry testing and/or myocardial perfusion scintigraphy) were included in the study. According to the Sphygmocor Clinical Guide instructions, patients with atrial fibrillation and aortic valve stenosis were excluded, as these two entities cause unreliable readings [23]. Patients with active malignancies and pregnant patients were excluded from the study as well. Patients were not withdrawn from regular medication. ABI, cfPWV and PWA measurements were performed at the Department of Dialysis, before planned coronary angiography.

The study was approved by the National Ethics Committee (N°0120-32/2017/4) and adhered to the Declaration of Helsinki. All patients gave written informed consent. The study was conducted according to Good Clinical Practice standards.

The following data were collected: medical history along with prescribed medications, body mass index (BMI), atherosclerosis risk factors and comorbidities (arterial hypertension, diabetes mellitus, heart failure, dyslipidemia, smoking, PAD, CKD), ABI, and arterial stiffness measurements. To perform basic laboratory tests, blood was drawn from a peripheral vein. Glomerular filtration rate was estimated (eGFR) by using the Chronic Kidney Disease Epidemiology Collaboration (CKD-EPI 2009 creatinine) equation.

### Ankle-brachial index measurement

ABI was measured using previously validated, automated, non-invasive waveform analysis device (MESI®, Slovenia) [24]. To reduce interobserver variability, all the ABI and arterial stiffness measurements were performed by two medical doctors. Every patient was instructed to lay still for five minutes before the measurements were obtained. Blood pressure was measured in the supine position, simultaneously on the brachial part of the right arm and calves of both legs. ABI was calculated automatically as the ratio of the highest ankle systolic blood pressure divided by the highest brachial systolic blood pressure. Every patient had three ABI measurements on each side. The average ABI value was taken as the study parameter.

### Pulse wave analysis and carotid-femoral pulse wave velocity measurements

For the assessment of arterial stiffness, applanation tonometry of the radial, carotid and femoral artery was used (SphygmoCor, AtCor Medical, Sydney, Australia). All the measurements were performed between 10 and 12 AM on weekdays. Before the measurement, subjects were under similar conditions (abstained from coffee, cigarettes, heavy meals, and exercise for at least 12 h prior). Each patient waited in a supine position for at least five minutes in a quiet room before the measurements were taken. During the measurements, all the cellphones and other electronic gadgets were turned off to prevent any distortions or errors in obtaining the data. Subtracted carotid-femoral distance was used [(sternal-femoral) − (carotid-sternal)] [25]. A validated transfer function was used to generate the corresponding aortic pressure waveform. Several PWA parameters were obtained. Pulse pressure (PP) was defined as the difference between systolic and diastolic blood pressure. The augmentation pressure (AP) was defined as the difference between the second and first systolic peak. Mean arterial pressure (MAP) was calculated by doubling the diastolic blood pressure, adding systolic pressure and then dividing the composite sum by three. The augmentation index (AIx), an indirect measure of arterial stiffness, was calculated as the ratio between AP and PP. The ejection duration (ED) was defined as the duration of left ventricular systolic ejection. Subendocardial blood supply as a parameter evaluating the risk of myocardial ischemia was expressed with subendocardial viability ratio (SEVR), the ratio between the diastolic and systolic time index [21]. AIx was corrected for a heart rate of 75/minute allowing us to compare patients with different heart rates (AIx@75). The following values for quality indices were considered acceptable: operator index ≥ 80%, average pulse height ≥ 80%, pulse height variation ≤ 5%, diastolic variation ≤ 5%.

Pulse wave distance between the carotid and femoral artery divided by pulse transit time (measured by electrocardiographic monitoring) was defined as cfPWV. Each patient had three optimal cfPWV measurements (with a standard deviation of less than 10%), the average value of cfPWV was then calculated and taken as the study parameter.

### Statistical analysis

Statistical analysis was performed with the Statistical Package for Social Sciences version 22.0 (SPSS Inc, Chicago, IL, USA). Basic descriptive statistics were used for continuous variables (mean ± standard deviation (SD)). For categorical variables, frequencies and percentages were used. The distribution of continuous variables was tested by using graphical methods (the normal probability plots, quantile–quantile plots) and by using the Shapiro–Wilk test. Due to non-normal distribution of our variables, non-parametric tests, such as the one-way analysis of variance on ranks (ANOVA), Chi-square test and Spearman's correlation coefficient were used to compare different variables. Multiple regression analysis was performed with arterial stiffness parameters which showed a statistically significant correlation with ABI (SEVR and cfPWV). The goal was to determine the impact of traditional atherosclerosis risk factors (age, diabetes, smoking status, eGFR, BMI, arterial hypertension, gender, and dyslipidemia), MAP and ABI on dependent variables, such as SEVR and cfPWV. For all tests, a *p* value of < 0.05 was statistically significant.

## Results

One hundred and twenty-three patients, aged 65.0 ± 9.4 years (range 27–82 years) were included in the study. The majority of included patients were male (n = 78, 63.4%). Average BMI was 28.6 ± 4.3 kg/m^2^ (range 18.9–42.5 kg/m^2^). Mean serum creatinine was 90.9 ± 67.4 μmol/L (range 49–666 μmol/L) and mean eGFR was 76.1 ± 16.9 ml/min/1.73 m^2^ (range 6–90 ml/min/1.73 m^2^). Mean ABI was 1.04 ± 0.12 (range 0.76–1.31). Seven patients (5.7%) had a mean ABI of less than 0.9. Most common comorbidities were arterial hypertension (n = 98, 79.7%), dyslipidemia (n = 69, 56.1%), diabetes mellitus type 2 (n = 26, 21.1%), heart failure (n = 14, 11.4%), previously known CKD (n = 8, 6.5%) and previously known PAD (n = 6, 4.9%). All of the patients with previously diagnosed PAD experienced mild symptoms of PAD (Rutherford grade I). None of the included patients were on the renal replacement therapy with hemodialysis or peritoneal dialysis or had a history of kidney transplantation. One included patient had diabetes mellitus type 1 (0.8%). The majority of patients were either previous smokers (n = 45, 36.6%) or non-smokers (n = 52, 42.3%), 19 patients (15.4%) were active smokers. For seven patients (5.7%) no data on smoking status was obtained.

Patient characterics, demographic data and descriptive statistics are presented in Table 1. The mean values of ABI, MAP and arterial stiffness parameters are shown in Table 2.

**Table 1:** Patient characteristics, demographic and descriptive data

Patient characteristic	Number of patients (%)	Patient characteristic	Mean ± SD
Male	78 (63.4)	BMI (kg/m^2^)	28.6 ± 4.3
Female	45 (36.6)	Total cholesterol (mmol/L)	4.6 ± 1.1
Arterial hypertension	98 (79.7)	LDL (mmol/L)	2.8 ± 1.0
Diabetes mellitus	27 (21.9)	HDL (mmol/L)	1.4 ± 0.4
Type 1	1 (0.8)	TG (mmol/L)	1.9 ± 1.0
Type 2	26 (21.1)	Creatinine (*µ*mol/L)	90.9 ± 67.4
Active smokers	19 (15.4)	eGFR (ml/min/1.73 m^2^)	76.1 ± 16.9
CKD	8 (6.5)	NT-proBNP (pmol/L)	57.8 ± 193.1
Heart failure	14 (11.4)	Homocysteine (*µ*mol/L)	14.2 ± 5.8
PAD	6 (4.9)	Cystatin C (mg/L)	1.1 ± 0.7
Dyslipidemia	69 (56.1)	CRP (mg/L)	5 ± 6.9
CAD	81 (65.9)	Hemoglobin (g/L)	140 ± 13.5
1-vessel CAD	21 (17.1)	Troponin I (*µ*g/L)	0.04 ± 0.15
2-vessel CAD	24 (19.5)	Uric acid (mmol/L)	332.2 ± 87.1
3-vessel CAD	36 (29.3)	Lp(a) (mg/L)	0.3 ± 0.4

*CKD* chronic kidney disease, *PAD* peripheral arterial disease, *CAD* coronary artery disease, *BMI* body mass index, *LDL* low density lipoprotein, *HDL* high density lipoprotein, *TG* triglycerides, *eGFR* estimated glomerular filtration rate, *NT-proBNP* N-terminal pro-brain natriuretic peptide, *CRP* C-reactive protein, *Lp(a)* lipoprotein(a), *SD* standard deviation

**Table 2:** Mean values of ABI, MAP, PWA parameters and cfPWV

Study parameter	Mean ± SD	Range
ABI	1.05 ± 0.11	0.77–1.31
SEVR (%)	166.6 ± 32.7	99–260
cfPWV (m/s)	10.3 ± 2.4	6.5–16.8
AIx (%)	29.0 ± 9.6	6–50
AIx@75 (%)	25.7 ± 8.5	6–50
AP (mmHg)	15.1 ± 8.7	2–47
ED (ms)	33.4 ± 4.0	24–44
PP (mmHg)	47.7 ± 15.0	17–94
Aortic systolic pressure (mmHg)	127.1 ± 21.1	98–202
Aortic diastolic pressure (mmHg)	77.8 ± 12.7	44–108
Mean MAP (mmHg)	94.1 ± 13.6	61.7–139.3
Heart rate (beats per minute)	68.3 ± 11.7	48–108

*ABI* ankle-brachial index, *MAP* mean arterial pressure, *PWA* pulse wave analysis, *cfPWV* carotid-femoral pulse wave velocity, *SD* standard deviation, *SEVR* subendocardial viability ratio, *Aix* augmentation index, *AIx@75* augmentation index adjusted to heart rate 75 beats per minute, *AP* augmentation pressure, *ED* ejection duration, *PP* pulse pressure

Most commonly prescribed medications were acetilsalycilyc acid (n = 106, 86.9%), statins (n = 92, 75.4%), beta blockers (n = 84, 68.9%), angiotensin-convertase inhibitors (n = 71, 58.2%), diuretics (n = 46, 37.7%), calcium channel blockers (n = 29, 23.8%), angiotensin receptor blockers (n = 22, 18.0%), metformin (n = 21, 17.2%), nitrates (n = 18, 14.8%) and alfa blockers (n = 17, 13.9%).

42 patients (34.1%) had a normal coronary angiogram, showing no atherosclerotic changes on the major coronary arteries (Table 3). The rest of the patients (n = 81, 65.9%) had CAD. Most of them had three-vessel CAD (n = 36, 29.3%), 24 patients (19.5%) had two-vessel CAD and 21 patients (17.1%) had one-vessel CAD. Interestingly, patients with zero- and three-vessel CAD had statistically significantly lower values of SEVR (159.5 ± 32.9% and 158.1 ± 31.5%) compared to those with one- and two-vessel CAD, respectively (181.0 ± 35.2% and 166.8 ± 27.8%; *p* = 0.048). Higher cfPWV values were observed in patients with zero- and three-vessel CAD compared to one- and two-vessel CAD as well (10.5 ± 3.2/10.7 ± 2.7 m/s vs 9.6 ± 2.1/10.4 ± 2.4 m/s), but the finding was not statistically significant (*p* = 0.487). Patients with zero- and three-vessel CAD had statistically significant higher MAP compared to those with one- and two-vessel CAD (97.3 ± 11.6/96.0 ± 18.6 vs 88.0 ± 9.2/92.1 ± 9.7, *p* = 0.043). None of the other arterial stiffness indices showed any correlation with CAD. Patients without CAD had more commonly prescribed beta blockers (21/42, 50.0%) compared to other patients (one-vessel CAD 4/21, 19%; two-vessel CAD 7/24, 29.2%; three-vessel CAD 7/36, 19.4%; *p* < 0.001). A similar trend was observed for angiotensin-convertase inhibitors (zero-vessel CAD 24/42, 57.1%; one-vessel CAD 4/21, 19.0%; two-vessel CAD 8/24, 33.3%; three-vessel CAD 16/36, 44.4%; *p* = 0.002). There were no differences between the groups in other prescribed medications.

**Table 3:** The characteristics of patients with different degrees of CAD

	No CAD (n = 42, 34.1%)	One-vessel CAD (N = 21, 17.1%)	Two-vessel CAD (N = 24, 19.5%)	Three-vessel CAD (n = 36, 29.3%)	*p*
Age (years)	62.5 ± 10.8	63.9 ± 9.2	65.7 ± 9.1	66.7 ± 7.8	0.250
Male gender	17 (40.5%)	14 (66.7%)	20 (83.3%)	27 (75.0%)	< **0.001**
BMI (kg/m^2^)	28.0 ± 4.3	28.2 ± 3.9	29.2 ± 3.9	28.6 ± 5.0	0.748
eGFR (ml/min/1.73 m^2^)	77.7 ± 20.6	78.2 ± 13.8	72.7 ± 17.3	73.3 ± 15.5	0.491
ABI	1.1 ± 0.1	1.0 ± 0.1	1.1 ± 0.1	1.0 ± 0.1	0.170
Active smoking	8 (19.0%)	7 (33.3%)	4 (16.7%)	3 (8.33%)	0.071
Arterial hypertension	26 (61.9%)	18 (85.7%)	24 (100%)	30 (83.3%)	0.110
Diabetes	6 (14.3%)	3 (14.3%)	5 (20.8%)	13 (36.1%)	0.140
MAP (mmHg)	97.3 ± 11.6	88.0 ± 9.2	92.1 ± 9.7	96.0 ± 18.6	**0.043**
Dyslipidemia	14 (33.3%)	16 (76.2%)	18 (75.0%)	21 (58.3%)	0.057
SEVR (%)	159.5 ± 32.9	181.0 ± 35.2	166.8 ± 27.8	158.1 ± 31.5	**0.048**
cfPWV (m/s)	10.5 ± 3.2	9.6 ± 2.1	10.4 ± 2.4	10.7 ± 2.7	0.487
AIx (%)	28.5 ± 11.1	30.3 ± 8.4	28.4 ± 8.8	28.7 ± 10.6	0.902
AIx@75	26.9 ± 12.2	25.6 ± 8.9	25.5 ± 7.5	25.8 ± 8.9	0.932
AP (mmHg)	14.2 ± 7.4	13.3 ± 5.6	14.9 ± 7.1	16.6 ± 11.0	0.469
ED (ms)	34.7 ± 3.8	32.5 ± 3.6	33.3 ± 4.0	34.1 ± 4.6	0.187
PP (mmHg)	47.0 ± 14.7	43.1 ± 13.5	48.4 ± 10.7	50.7 ± 18.1	0.293
HR (beats/minute)	70.1 ± 10.5	65.1 ± 8.8	69.0 ± 12.4	69.0 ± 12.4	0.390

For all tests, a *p*-value of < 0.05 was statistically significant which are indicated in bold

*CAD* coronary artery disease, *BMI* body mass index, *eGFR* estimated glomerular filtration rate, *ABI* ankle-brachial index, *MAP* mean arterial pressure, *SEVR* subendocardial viability ratio, *cfPWV* carotid-femoral pulse wave velocity, *Aix* augmentation index, *AIx@75* augmentation index adjusted for heart rate 75/minute, *AP* augmentation pressure, *ED* ejection duration, *PP* pulse pressure, *HR* heart rate

Gender analysis revealed that men had a higher incidence of macrovascular CAD (61/81, 75.3%; *p* =  < 0.001). The majority of patients without CAD were female (25/42, 59.5%). There was no difference in ABI (*p* = 0.246), comorbidities or prescribed medications between male and female patients with different degrees of CAD.

Spearmann's correlation test showed a statistically significant correlation between ABI and SEVR (r = 0.196; *p* = 0.037) and between ABI and cfPWV (r = − 0.320; *p* ≤ 0.001). An important association was found between MAP and several arterial stiffness parameters, such as SEVR (r = − 0.223; *p* = 0.013), cfPWV (r = 0.296; *p* < 0.001), AIx (r = 0.252; *p* = 0.005), AIx@75 (r = 0.365; *p* < 0.001), AP (r = 0.338; *p* < 0.001), ED (r = 0.250; *p* = 0.005) and PP (r = 0.302; *p* < 0.001). MAP showed no statistically significant correlation with ABI.

Multiple regression analysis with cfPWV as dependent variable and age, gender, diabetes, smoking status, BMI, eGFR, ABI, MAP, dyslipidemia and arterial hypertension as independent variables, showed a statistically significant association between cfPWV and ABI (β = − 0.210; *p* = 0.003) (Table 4). An association was found between cfPWV and age (β = 0.113; *p* < 0.001), cfPWV and BMI (β = 0.206; *p* < 0.001) and between cfPWV and MAP (β = 0.064; *p* < 0.001) as well. The same multiple regression analysis model was used for SEVR as dependent variable. No statistically significant association between SEVR and ABI was found. An association was found between SEVR and age (β = − 0.206; *p* = 0.031), male gender (β = 0.429; *p* < 0.001), BMI (β = − 0.195; *p* = 0.028) and MAP (− 0.246; *p* = 0.005).

**Table 4:** The impact of several traditional atherosclerosis risk factors on cfPWV and SEVR

Independent variables	cfPWV^a^				SEVR^b^			
	β	SE	t	*p*	β	SE	t	*p*
Age (years)	0.113	0.021	5.351	**< 0.001**	− 0.206	0.315	− 0.654	**0.031**
Male gender	0.436	0.371	1.174	0.243	0.429	0.284	1.150	**< 0.001**
Active smoking	0.120	0.228	0.528	0.599	− 0.241	0.166	− 1.452	0.560
Arterial hypertension	0.177	0.479	0.244	0.808	− 0.349	0.642	− 0.544	0.357
Diabetes mellitus	0.561	0.446	1.258	0.211	− 0.431	0.219	− 1.968	0.746
Dyslipidemia	0.072	0.381	0.188	0.851	− 0.118	0.214	− 0.514	0.709
eGFR (ml/min/1.73 m^2^)	− 0.007	0.011	− 0.649	0.518	0.099	0.170	0.582	0.562
ABI	− 0.210	1.601	− 3.038	**0.003**	0.218	0.135	1.615	0.146
BMI (kg/m^2^)	0.206	0.044	4.684	**< 0.001**	− 0.195	0.654	− 0.298	**0.028**
MAP (mmHg)	0.064	0.014	4.714	**< 0.001**	− 0.246	0.202	− 1.218	**0.005**

For all tests, a *p*-value of < 0.05 was statistically significant which are indicated in bold

*cfPWV* carotid-femoral pulse wave velocity, *SEVR* subendocardial viability ratio, *SE* standard error, *eGFR* estimated glomerular filtration rate, *ABI* ankle brachial index, *BMI* body mass index, *MAP* mean arterial pressure

^a^Dependent variable cfPWV (R = 0.749, R^2^ = 0.561, adjusted R^2^ = 0.518)

^b^Dependent variable SEVR (R = 0.556, R^2^ = 0.309, adjusted R^2^ = 0.242)

## Discussion

According to the results of our study, lower ABI is associated with increased cfPWV and decreased SEVR. The association between ABI and cfPWV is present even when confounded for several traditional atherosclerosis risk factors (dyslipidemia, arterial hypertension, diabetes, kidney function and smoking status). It is, however, pivotal to recognize the significant impact of higher BMI, advanced age and higher MAP on increased cfPWV and decreased SEVR as well. Patients with zero- and three-vessel CAD had importantly lower SEVR and slightly higher cfPWV values, compared to those with one- and two-vessel wall CAD, but also had higher MAP and different prescribed medications (more beta blockers and angiotensin-convertase inhibitors). No association was found between different degrees of CAD and ABI.

Increased arterial stiffness is associated with several pathological states. According to studies, increased AIx, AP, and AIx@75 are independent risk markers for premature CAD [26, 27]. An important relation between the left main coronary artery disease and increased PWV has previously been established [28]. Not only macrovascular but also microvascular CAD and reduced coronary blood flow reserve have been observed in patients with increased vessel wall stiffness [29]. Cerebrovascular disease, dementia, and cognitive impairment are more prominent as well [30–32]. Many studies have been performed on patients with dyslipidemia, diabetes, and CKD, all showing increased vessel wall stiffness and a higher incidence of major adverse cardiovascular events in these cohorts [33–36].

The number of studies on patients with PAD, however, is small. Mosimann et al. performed a study on 65 stable PAD patients [19]. They found a statistically significant negative relationship between ABI, AIx and SEVR values, pointing out a likely connection between peripheral perfusion, central aortic pressure augmentation and subendocardial perfusion [22]. In a cross-sectional study by Zahner et al., patients with PAD (n = 101) were compared to controls without known atherosclerotic disease (n = 33) in PWA parameters. Higher AIx was connected with PAD according to their results [37]. In a study by Del Brutto et al., even slightly reduced ABI was associated with increased PP, which is also a sign of increased arterial stiffness [38]. We found that the gold standard of central arterial stiffness—cfPWV is higher in patients with reduced ABI and higher age/MAP/BMI, but is not associated with several other traditional atherosclerosis risk factors and comorbidities. It is known that age-related fragmentation of elastic fibers, alterations in vascular tone and higher blood pressure leads to higher cfPWV values in older patients [39]. The data on the association between BMI and arterial stiffness parameters is, on the other hand, contradictory. In a study by Tang et al., the authors found a significant increase in ankle-brachial pulse wave velocity (abPWV) in patients with higher BMI values in studied cohort [40], while Rodriques et al. failed to prove an independent association between cfPWV and BMI [41]. The authors Desamericq et al. performed a study on 2.034 patients in which they found no relationship between cfPWV and BMI as well [42]. In our study, the association between higher BMI, increased arterial stiffness and reduced subendocardial perfusion is present and could be explained by the fact that the included patients had a high suspicion of CAD and were therefore hospitalized for elective coronary angiography. This leads to a bias in the studied group, which is the reason why further studies should be done on study groups that are better representative of the general population.

Mean values of ABI in our patients were in the normal range, which leads us to believe that even small reductions in ABI have an impact on pulse wave propagation. Interestingly, compared to other studies, no link between ABI, AIx, AIx@75 or other PWA parameters was found. This could be due to higher mean ABI values in our patients compared to other studies.

Besides atherosclerosis, it appears that arterial stiffness by changes in pulse wave propagation causes a reduction in subendocardial perfusion. The terminal abdominal aorta acts as an important pulse wave reflection and buffering site. In subjects with PAD, the pressure waves travel through the arterial system more quickly because of the reduced arterial lumen. This causes superimposition of the forward and backward waves during systole, a prolongation in systole and an increase in the afterload, reflecting in increased cfPWV and reduced SEVR (as observed in our study) [43]. Indirect evidence of this has been shown by a study of subjects after traumatic limb amputation. Such subjects display a high incidence of systolic hypertension and increased arterial stiffness, together with a change in pulse wave propagation due to shorter arterial system [44].

All the above mentioned data show the importance of ABI in understanding the interplay between structural and functional vessel wall changes in patients with atherosclerosis. This is especially evident from a study by Jacomella et al. in which they compared conservatively treated PAD patients (n = 48) to age- and sex-matched PAD patients treated with endovascular lower-extremity revascularization procedure (n = 61) [45]. They found a reduction in AIx values after endovascular treatment, indicating an impact of revascularization on pulse-wave function [45]. PWA markers, such as AIx provide important information on wave reflection characteristics but are only indirect surrogate markers of arterial stiffness [46]. In comparison, in our study only 6 patients (4.9%) had a previously diagnosed PAD (all were Rutherford grade I) and only 7 out of 123 patients (5.7%) had a mean ABI value lower than 0.9, which is an important methodological difference compared to other studies as the number of PAD-naive patients in our study was much higher. Besides PWA measurements, a direct arterial stiffness measurement (cfPWV) was obtained as well, enabling us to study the immediate link between ABI-assessed peripheral perfusion, central arterial stiffness, pulse wave propagation and ultimately CAD.

Surprisingly, even though the included patients had a high clinical suspicion of myocardial ischemia and a previously positive either cycle ergometry testing and/or myocardial perfusion scintigraphy, coronarography showed a relatively large subgroup of patients without macrovascular CAD (n = 42, 34.1%). This subgroup of patients had values of SEVR and cfPWV comparable to those with three-vessel CAD. Patients in the one- and two-vessel CAD group faired better in arterial stiffness parameters and they did not differ in ABI values or comorbidities. It is important to note that patients in the zero- and three-vessel CAD had slightly higher MAP values and due to unknown reason, a higher percentage of prescribed beta-blockers and angiotensin-convertase inhibitors, both of which can lead to changes in cfPWV and SEVR values [47]. Another explanation for this (especially in those without macrovascular CAD) could be in microvascular dysfunction [48], but this was not objectively tested in the parent study. Further studies are needed to confirm this hypothesis.

Looking at the results of our study, male gender was associated with higher SEVR. Several studies have shown that women are more likely to develop left ventricular hypertrophy and heart failure in the presence of myocardial ischemia. This gender difference is not completely explained by the difference in risk factors and/or treatment regimen. Multiple gender-related differences were observed in central systolic pressure augmentation and in SEVR as well, implying a gender-based difference in vessel wall hemodynamics [49]. A possible explanation for these findings could be a higher incidence of endothelial dysfunction often found in women, which can cause a decrease in SEVR [50]. Since all the included patients had a high clinical suspicion of myocardial ischemia due to a positive non-invasive testing, a higher incidence of women without macrovascular CAD confirms the idea of more pronounced functional changes and endothelial dysfunction in this sub-group of patients. To accurately determine the impact of gender on SEVR, especially in PAD patients, further studies need to be conducted.

Reduced ABI and increased arterial stiffness are pathophysiologically linked, not only through atherosclerosis, but through several other important pathophysiologic mechanisms. From the results of our study it appears that reduced peripheral perfusion impacts coronary perfusion through changes in pulse wave reflection and blood flow. An important cause of concern in this population is not only limb ischemia but also an increased likelihood of cardiovascular events and higher mortality. Arterial stiffness, determined by non-invasive applanation tonometry, can be useful in risk-stratifying patients with PAD and in optimizing the correct treatment regimen. Early revascularization therapy could prevent the detrimental impact of earlier pulse wave reflection to the myocardium and therefore lead to a reduction in cardiovascular events in this population. More studies on how to implement PWA and cfPWV measurements in the routine assessment of patients with PAD should be done.

## Limitations and strengths

An important limitation of our study is the cross-sectional design and small sample size. Only Caucasian patients were included in the study which is an important limitation—it is known there are differences in atherosclerosis and arterial stiffness parameters between races [51].

It would be interesting to see an interventional study on changes in cfPWV and SEVR values after performing a revascularization procedure in patients with PAD. A decrease in cfPWV and an increase in SEVR would further confirm our hypothesis that narrowing of the peripheral arteries causes earlier retrograde pulse wave propagation. Bigger studies should be done on patients with PAD to advance our understanding of blood flow and vessel wall stiffness.

An important strength of our study is the potential clinical implications it has—we believe that early treatment of patients with PAD is pivotal in reducing their cardiovascular risk. Risk stratification is an important area of research in this population.

## Conclusions

Reduced ABI is associated with increased cfPWV and decreased SEVR, indicating an important connection between peripheral arteries and coronary circulation. Pulse wave propagation is different in obstructed peripheral vessels, causing an earlier return of pulse wave towards the heart and impacting its workload and perfusion. Besides managing atherosclerosis risk factors, more attention should be given to arterial stiffness parameters as well as they can be an important risk stratification tool of patients with PAD.

## Data Availability

If needed, additional data and materials that are connected to the presented study and article are available.
